# Supplementary material for: Identification of photoperiod-induced specific miRNAs in the adrenal glands of Sunite sheep (Ovis aries)
Source: Front Vet Sci. 2022 Jul 22;9:888207. doi: 10.3389/fvets.2022.888207 (PMC9354845; doi:10.3389/fvets.2022.888207)
Supplement: Supplementary file 1 [file Data_Sheet_1.ZIP › Supplementary file/miRDeep2 file.zip!/miRDeep2 file/Know_Structure/LP42A1/oar-mir-369.pdf]

miRBase precursor : oar-mir-369
Total read count : 8345
oar-miR-369-5p read count : 760
oar-miR-369-3p read count : 7583
remaining reads : 2

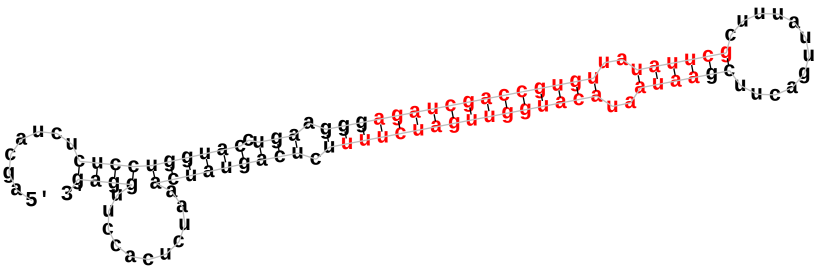

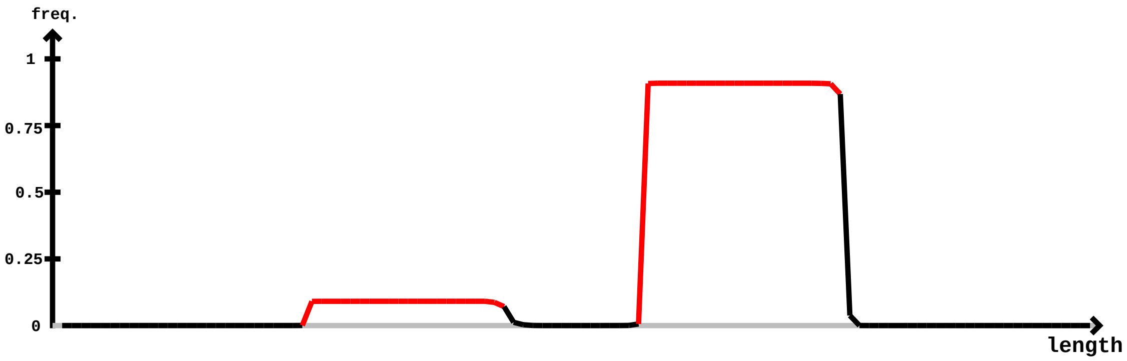

oar-miR-369-5p

oar-miR-369-3p

```
5'- agcaucucuccugguaccugaagggagaucgaccguguuauauucgcuuuauugacuucgaauaauacaugguugaucuuuucucaguaucaaaucucaccuuggag   -3'    exp
    .......((((((((((.(((.((((((((((((((((..((((((..............))))))..)))))))))))))))).))))))))).........))))    reads    mm    sample
    .....cucuccugguaccugaaggg..................................................................................    1    0    seq
    ........................gagaucgaccguguuauauucg.............................................................    2    0    seq
    .........................agaucgaccguguuauauu...............................................................    35    0    seq
    .........................agaucgaccguguuauauuc..............................................................    131    0    seq
    .........................agaucgaccguguuauauucg.............................................................    488    0    seq
    .........................agaucgaccguguuauauucgc............................................................    75    0    seq
    .........................agaucgaccguguuauauucgcu...........................................................    23    0    seq
    .........................agaucgaccguguuauauucgcuu..........................................................    3    0    seq
    ..........................gaucgaccguguuauauucg.............................................................    2    0    seq
    ..............................gaccguguuauauucgc............................................................    1    0    seq
    ........................................................uucgaauaauacaugguugaucu............................    1    0    seq
    ..........................................................cgaauaauacaugguugaucuu...........................    1    0    seq
    ..........................................................cgaauaauacaugguugaucuuu..........................    2    0    seq
    ...........................................................gaauaauacaugguugaucuu...........................    8    0    seq
    ...........................................................gaauaauacaugguugaucuuu..........................    39    0    seq
    ............................................................aauCauacaugguugau..............................    1    1    seq
    ............................................................aauaauacaugguugauc.............................    5    0    seq
    ............................................................aauaauacaugguugaucu............................    12    0    seq
    ............................................................aauaauacaugguugaucuu...........................    312    0    seq
    ............................................................aauaauacaugguugaucuuu..........................    6882    0    seq
    ............................................................aauaauaAaugguugaucuuu..........................    1    1    seq
    ............................................................aauaauacaugguugaucuuuu.........................    311    0    seq
    .............................................................auaauacaugguugaucuuu..........................    5    0    seq
    .............................................................auaauacaugguugaucuuuu.........................    2    0    seq
    .............................................................auaauacaugguugaucuuuuc........................    2    0    seq
```
